# Group association and vocal behaviour during foraging trips in Gentoo penguins

**DOI:** 10.1038/s41598-017-07900-7

**Published:** 2017-08-17

**Authors:** Noori Choi, Jeong-Hoon Kim, Nobuo Kokubun, Seongseop Park, Hosung Chung, Won Young Lee

**Affiliations:** 1 Division of Polar Life Sciences, Korea Polar Research Institute, Incheon, Republic of Korea; 2 School of Biological Sciences, University of Nebraska, Lincoln, United States of America; 3 National Institute of Polar Research, Tokyo, Japan; 4 Division of Life Sciences, Incheon National University, Incheon, Republic of Korea

## Abstract

In contrast to their terrestrial call, the offshore call of penguins during their foraging trips has been poorly studied due to the inaccessibility of the foraging site—the open ocean—to researchers. Here, we present the first description of the vocal behaviour of penguins in the open ocean and discuss the function of their vocal communication. We deployed an animal-borne camera on gentoo penguins (*Pygoscelis papua*) and recorded their foraging behaviour during chick guarding. From the video recordings, we collected 598 offshore calls from 10 individuals in two breeding seasons (2014–2015 and 2015–2016), and we analysed the acoustic characteristics and behavioural contexts of these calls, including diving patterns, group association events, and foraging behaviour. The offshore calls varied in their dominant frequency and length, and penguins produced calls of different lengths in succession. Group associations were observed within one minute following an offshore call in almost half of the instances (43.18%). Penguins undertook dives of shallower depths and shorter durations after producing an offshore call than those before producing an offshore call. Our findings show that penguins may use vocal communication in the ocean related with group association during foraging trips.

## Introduction

Group living provides many potential benefits, including reduced predation risk^1^, increased foraging efficiency^2^, and tolerance to harsh environments^3^. Many avian species breed in large colonies and gain benefits from group behaviours, including group foraging^2^ and group defence, such as mobbing behaviour^4^. When engaged in group behaviours, individuals use a diverse repertoire of calls^5^. Calls may enable group-living birds to exchange information, gather group members, and coordinate group movements across various contexts^5^.

Marler^5^ categorized avian calls into four functional types: 1) calls involved in maintaining group coherence, 2) calls involved in food sharing, 3) calls involved in hostile interactions, and 4) alarm calls. Birds produce contact calls^5, 6^, flight calls^7^, and roosting calls^5^ to maintain group association in place and time while they are foraging^6^, traveling^7^, or resting^5^. Food calls and begging calls are associated with food sharing. Food calls recruit other members to a food source^5, 8, 9^, and begging calls stimulate parents to feed their offspring^10, 11^. Hostile interactions among group members provoke aggressive calls^10^, which function to resolve conflicts over resources^5, 11^. Various types of alarm calls, such as distress calls^10^ and mobbing calls^12^, induce diverse defence mechanisms^5, 12^. Behavioural change concomitant with a call can provide insight into the function of group behaviour. For instance, the pied babbler (*Turdoides bicolor*), which is a colonial-breeding bird species, produces several types of calls that function in anti-predatory defence and increasing foraging efficiency at foraging sites^13–15^.

Penguins are good model species to study group living and vocal behaviour because these birds are colonial breeders^16^ and are highly dependent on vocal communication for group living. The vocal repertoires are used in various contexts, including sexual selection (Adélie penguins, *Pygoscelis adelie* ^17^), parent-offspring recognition (king penguins, *Aptenodytes patagonicus* ^18, 19^; Adélie penguins; and gentoo penguins, *Pygoscelis papua* ^20^), and territory defence (African penguins, *Pheniscus demersus* ^11^). Favaro *et al*.^11^ reported four types of calls produced by the African penguin: *contact calls*, *agonistic calls*, *display songs*, and the *begging moan*. The African penguin produces *contact calls* when it is isolated from other members. *Ecstatic and mutual display songs* are used in recognition of social bonds between mates or in parent-offspring relationships. *Agonistic calls* are presented with aggressive behaviours, and the *begging moan* is produced by chicks to induce their parents to regurgitate food. However, the inaccessibility of open ocean foraging sites of penguins to researchers has precluded the study of the vocal repertoires used during foraging trips, except for a few brief observations^21–23^. This inaccessibility hinders the study of not only penguin vocal behaviour but also the evolutionary significance of group foraging behaviour in penguins.

Many penguin species forage in groups (e.g., little penguins, *Eudyptula minor* ^23^ and Adélie penguins^24^), and two hypotheses have been proposed regarding the benefits of group foraging: the ‘anti-predation hypothesis’^25^ and the ‘increased foraging efficiency hypothesis’^26, 27^. The anti-predation hypothesis states that group foraging behaviour decreases predation pressure by two main effects: the vigilance effect^13, 15^ and the dilution effect^28, 29^. Takahashi *et al*.^25^ suggested that the group foraging behaviour of the Adélie penguin decreases predation pressure at a cost of reduced feeding time due to the synchronous ascending and descending with other group members. The increased foraging efficiency hypothesis states that group foraging increases foraging efficiency via information sharing^30–32^ and/or cooperative foraging^33^. Sutton *et al*.^23^ provided evidence that the group foraging of little penguins may increase the detection of small patches of highly mobile prey species. However, verification of these hypotheses has been hindered by the difficulty of conducting observations and manipulative experiments in the open ocean.

Recent advances in animal-borne bio-loggers enable the detailed investigation of foraging behaviour of diving seabirds in the open ocean^23, 24, 27, 34–36^. Using a video camera with a built-in microphone provides a unique opportunity to investigate the foraging behaviour of sea birds, which are largely dependent on acoustic communication^37^. Here, we used animal-borne cameras to investigate the foraging behaviour of the gentoo penguin at King George Island. We observed that the birds produce calls in the open ocean (hereafter referred to as “offshore calls”) during foraging trips.

The gentoo penguin, which is distributed from the sub-Antarctic islands to the Antarctic region, primarily preys in groups on krill and fish^25, 38–40^. Copeland observed that one or two individuals within a foraging group called on the surface of the ocean after diving^22^. In this study, we provide a detailed description of the offshore call of the gentoo penguin that is produced during foraging trips and analyse the relationship between the call and the behavioural context. We investigate the acoustic characteristics of the offshore call and discuss the functions and evolutionary benefits of group foraging.

## Results

**Figure 1:**
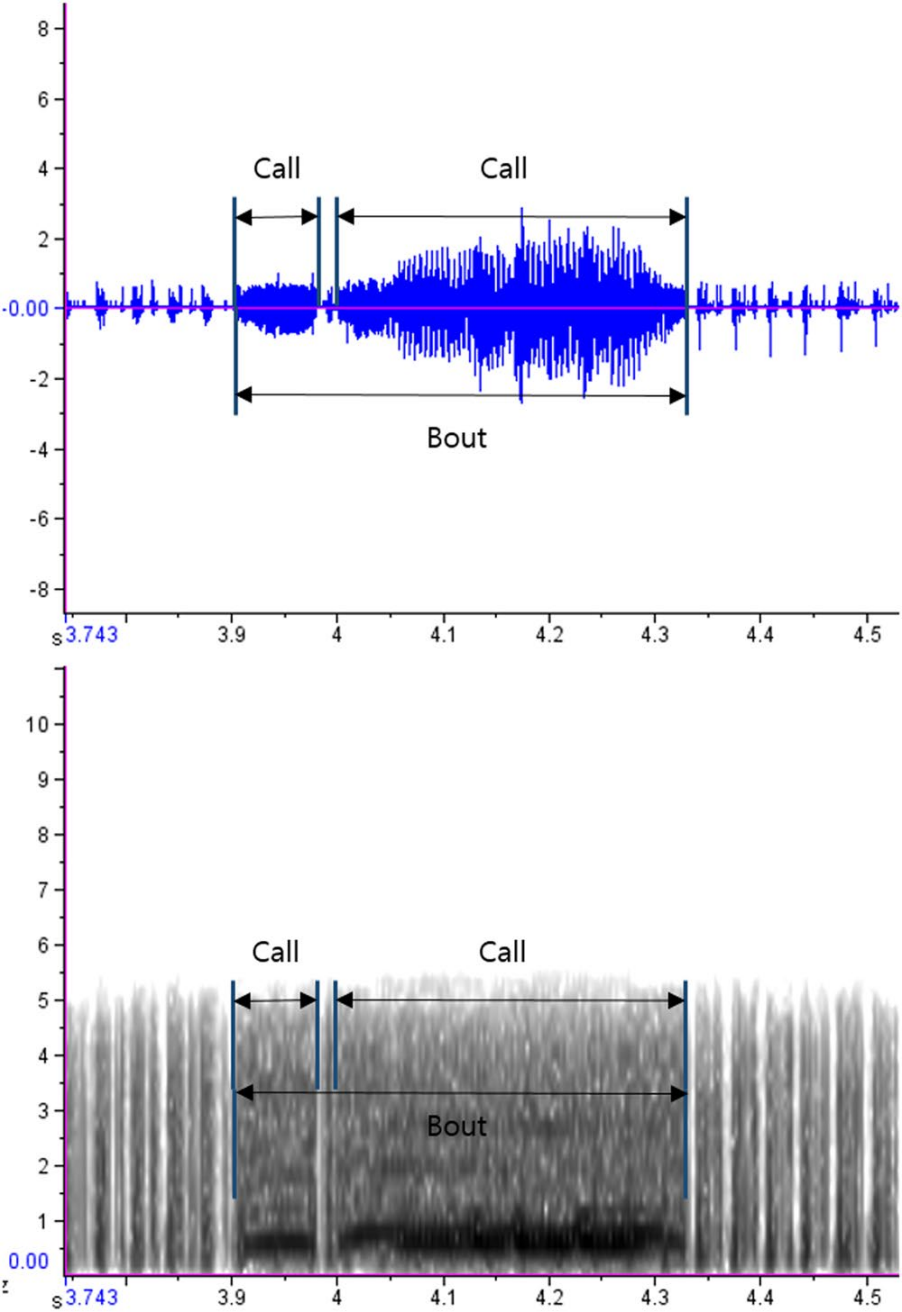
Oscillogram (top) and spectrogram (bottom) of a typical individual offshore call in the ocean (256-point fast Fourier transform, overlap 50%, Hanning window). The call comprises two notes that have different note durations.

**Figure 2:**
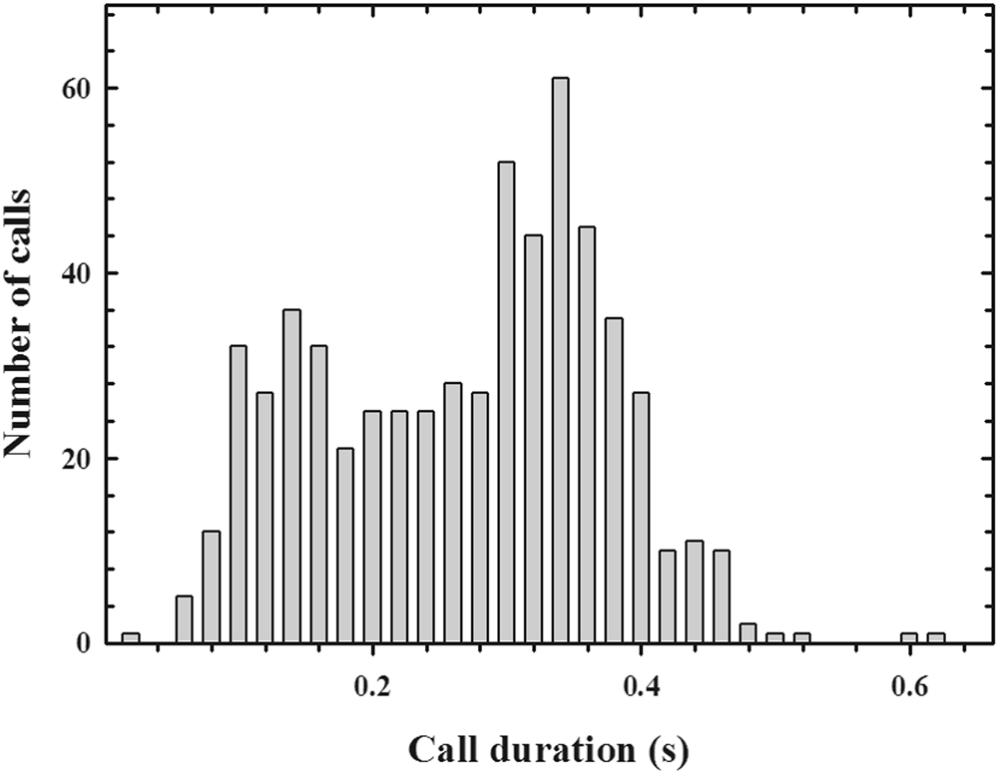
Histogram of note duration of recorded offshore calls (n = 597). The bin size is 0.02 seconds.

**Figure 3:**
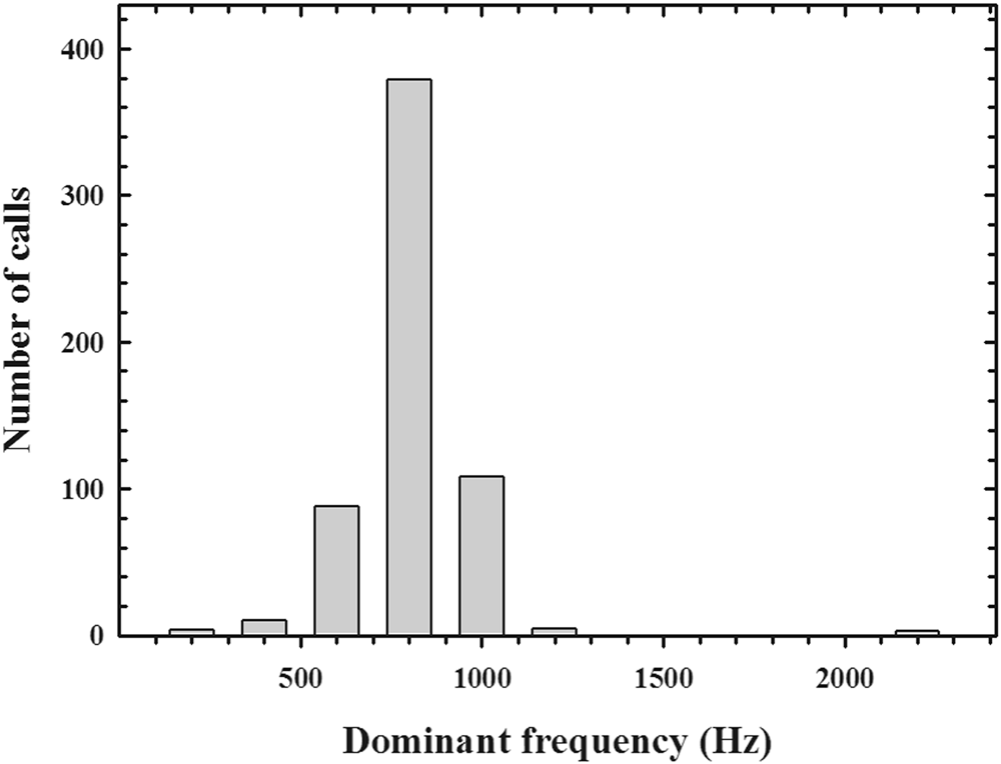
Histogram of dominant frequency of recorded offshore calls (n = 597). The bin size is 200 Hz.

**Figure 4:**
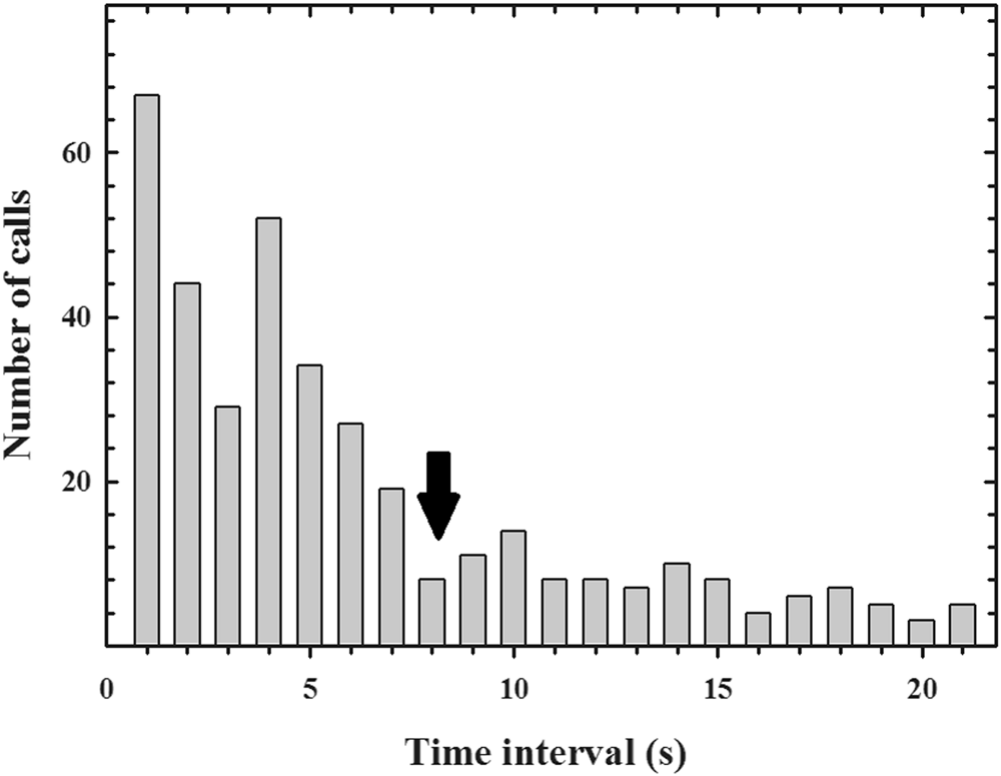
Distribution of the interval between individual offshore calls within 20 seconds. The arrow denotes the criterion (8 seconds) to integrate adjacent notes into a bout. The bin size is 1 second.

From the video recordings, we obtained 598 offshore calls (characterized in Fig. 1) from 10 individuals in two Antarctic summers during chick-guarding periods (2014–2015 and 2015–2016; Table 1). The average (±SD) call duration (CD) was 0.26 ± 0.11 seconds (Fig. 2) and the average dominant frequency (DF) of the offshore calls was 695.43 ± 155.10 Hz (Fig. 3). The distributions of CD and DF deviated from normal (CD: Kolmogorov-Smirnov with Lilliefors significance correction = 0.09, *p* < 0.001, *df* = 597; DF: Kolmogorov-Smirnov with Lilliefors significance correction = 0.32, *p* < 0.001, *df* = 597) (Figs 2 and 3, Table 2). We categorized all calls that were produced within an eight-second period as a “bout” (Fig. 4) to avoid repeated measures in the behavioural context analysis. A diving event ceased a calling activity on surface so we expected that the quantile in Fig. 4 may represent the average time span of one consecutive calling bout. A bout consisted of 1.930 ± 2.08 calls on average, and the average bout length was 2.88 ± 5.72 seconds.

**Table 1:** Recording time and the number of recorded offshore calls of the individuals used for analysis. Monitoring time was calculated by the proportion of trip duration recorded by camera to total trip duration recorded by accelerometer.

Individual	Sex	Date (MM/DD/YY)	Trip duration (h)	Monitoring time (%)	Number of calls	Number of bouts
1317	M	12/23/14	9.2	100	5	3
1322	F	12/25/14	8.0	75	34	12
1329	M	12/29/14	9.2	90	73	46
1335	M	12/30/14	13.1	60	81	26
1337	M	12/31/14	8.7	77	94	44
1340	F	01/03/15	12.9	82	117	51
1508	M	12/08/15	17.0	62	50	32
1509	M	12/08/15	9.7	89	90	61
1510	M	12/09/15	4.7	100	8	8
1516	M	12/21/15	9.3	100	43	25

**Table 2:** Kolmogorov-Smirnov test of call duration and dominant frequency of calling individuals. Normal distributions in each individual are denoted in bold. In two individuals (1317 and 1516) certain frequency values were repeated so it prevented to produce statistical results for testing dominant frequency.

ID	Call duration			Dominant frequency		
	D	df	*p*	D	df	*p*
1317	0.32	5	**0.11**	—	—	—
1322	0.16	34	0.03	0.497	34	<0.01
1329	0.09	73	**0.20**	0.499	73	<0.01
1335	0.25	81	<0.01	0.352	81	<0.01
1337	0.11	94	0.01	0.434	94	<0.01
1340	0.12	117	<0.01	0.332	117	<0.01
1508	0.21	50	<0.01	0.437	50	<0.01
1509	0.09	90	**0.06**	0.440	90	<0.01
1510	0.15	8	**0.20**	—	—	—
1516	0.14	43	0.04	0.395	43	<0.01
Total	0.09	597	<0.01	0.322	597	<0.01

**Figure 5:**
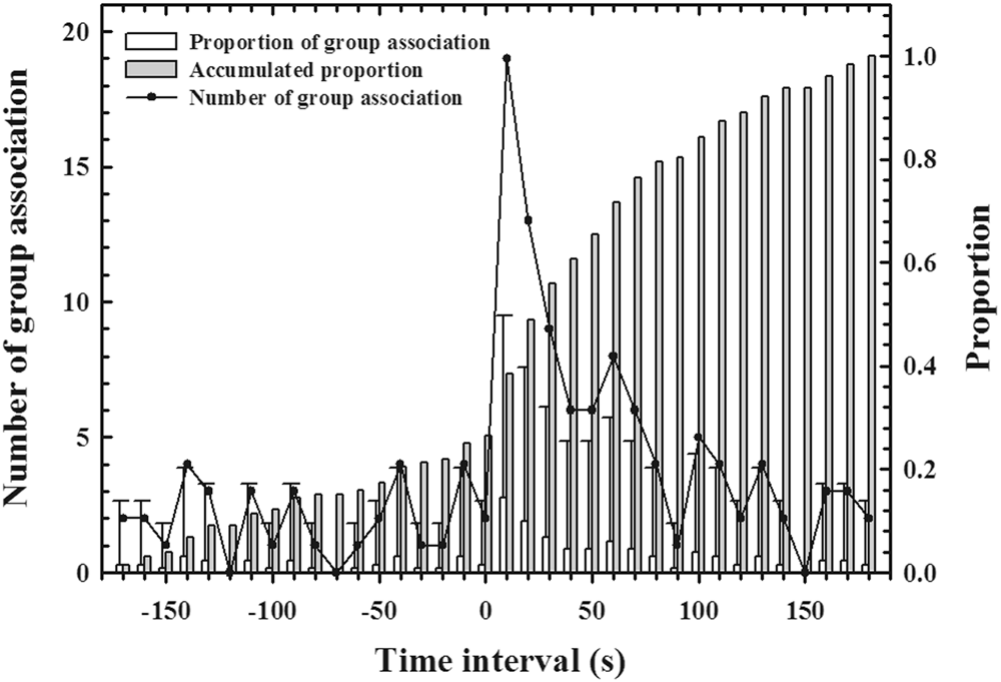
Distribution of group association within three minutes before and after a calling bout (n = 132). The error bars indicate the standard deviations of the mean value of the proportion of group associations.

When compared with the display songs with series of syllables of other gentoo penguins^20^, the offshore calls in this study had a simpler structure with only one or two calls and shorter duration (Table 3). Although the dominant frequency of the offshore calls is still within the range of the display song of gentoo penguins, it seem to be closer to the *contact calls* and *aggressive calls* of African penguins, which are uttered as single, short notes^11^ (Table 3). After the offshore calls were produced, penguins often moved unidirectionally, swimming near the surface (see Supplementary video S1). Almost half of the total number of group associations (47.74%) occurred within a minute after a vocal behaviour was performed (Fig. 5) and these penguins with group association exhibited shorter diving duration (N = 72, 104.78 ± 8.67 seconds) than those with no group associations (N = 83, 137.81 ± 8.18 seconds) (Student’s *t* test; *t*_153_ = 2.77, *p* = 0.01). Other dive parameters (maximum dive depth, average dive depth, dive type and prey capture rate) did not show significant changes (Student’s *t* test; all *p* > 0.08). Group association behaviour was observed in 132 of 308 bouts (42.86%).

**Table 3:** Comparison of the acoustic characteristics of penguin calls in this study and the two previous studies.

	This study	Jouventin and Aubin’s study^20^	Favaro *et al*.’s study^11^
Species	Gentoo	Gentoo	African penguin
Function	In discussion	Display song	Contact call
Call structure	One or two calls	Series of syllables	Single utterance
Call duration (s)	0.26 ± 0.11	1.16 ± 0.36	*contact call*; 0.58 ± 0.18 *aggressive call*; 0.44 ± 0.15
Dominant frequency (Hz)	695.43 ± 155.10	300–2500	*contact call*; 258 ± 34 *aggressive call*; 251 ± 36

Dive duration, maximum dive depth, and average dive depth significantly decreased after production of a calling bout within three minutes (Table 4). Gentoo penguins undertook shorter-duration (paired sample *t* test; *t* = 3.45, *p* < 0.001) and shallower dives (paired sample *t* test; *t* = 2.04, *p* = 0.04) after a calling bout than those before an offshore call. The ratio of exploratory dives to foraging dives (paired sample *t* test; *t* = 0.58, *p* = 0.56) and krill capture rate (paired sample *t* test; *t* = 1.10, *p* = 0.28) did not differ significantly between before and after a calling bout within three minutes.

**Table 4:** Paired sample *t* test of the difference in the parameters of penguin dives between before and after a calling bout within three minutes. Significant variables are denoted in bold.

Parameter	n	Before (Mean ± SD)	After (Mean ± SD)	*t*	*df*	*p* (two-tailed)
DD (s)	155	142.46 ± 73.22	122.47 ± 75.67	3.446	154	**0.001**
MDD (m)	155	56.78 ± 32.52	50.96 ± 35.30	2.474	154	**0.014**
ADD (m)	155	50.91 ± 30.18	46.04 ± 33.96	2.041	154	**0.043**
DT	80	0.97 ± 0.14	0.96 ± 0.14	0.584	79	0.561
PCR (number/s)	122	0.30 ± 1.45	0.12 ± 0.37	1.096	71	0.277

DD = Dive duration, MDD = Maximum dive depth, ADD = Average dive depth, DT = Dive type, PCR = Prey capture rate.

When comparing five diving parameters (dive duration (DD), maximum dive depth (MDD), average dive depth (ADD), dive type (DT) and prey capture rate (PCR)) between calling and non-calling individuals, no significant differences were found (Student’s *t* test; DD: *t*_18_ = −0.79, *p* = 0.44, MDD: *t*_*7.509*_ = −1.04, *p* = 0.33, ADD: *t*_*6.003*_ = 1.01, *p* = 0.35, DT: *t*_*6.099*_ = 1.00, *p* = 0.35, PCR: *t*_18_ = 0.78, *p* = 0.45).

## Discussion

We showed that gentoo penguins produced offshore calls and many individuals formed groups with the calls in the open ocean. These results generally support the hypothesis that the offshore call is related to group association during foraging trips. Thiebault *et al*.^37^ revealed that Cape gannets (*Morus capensis*) used vocal communication among individuals at sea, possibly to avoid collisions. In general, the penguin calls that we analysed were produced by lone individuals, and no vocal interactions with closely neighbouring individuals were recorded in our video-recordings. The absence of vocal interaction among neighbouring individuals may suggest that the function of the offshore call is more likely about group formation, not interaction among members in a pre-formed group^37^.

After producing offshore calls, the penguins undertook shallower, shorter dives than those before the call, and the video data showed that following their calls, the penguins travelled instead of lingering in a particular area (See Supplementary video S1). Moreover, although it was not possible to analyse detailed vocal exchanges due to the location of the microphone (attached snugly to the penguin’s back), calls made by other individuals (See Supplementary video S2) were also occasionally observed, which implies the occurrence of vocal interactions among multiple individuals. Considering the energetic cost of acoustic signalling, the shallower and shorter dives following a call could be related with production of offshore calls. If there is a trade-off between dive recovery and calling behaviour, it is possible that the calling individuals may incur a slight oxygen debt and accordingly it could affect dive dynamics.

The gentoo penguins did not show a significant difference in the proportion of foraging dives or prey capture rate between before and after producing an offshore call. These results suggest that the call may be a contact call rather than a food or recruitment call; however, an assessment of the abundance of food sources would be needed to draw a definitive conclusion.

We did not find any differences in diving behaviours between calling and non-calling individuals. However, it still remains unclear why some individuals did not produce any offshore calls. Our video-recording could cover only eight hours per individual so we are not able to generalize its foraging and vocal behaviour. We think that multiple recordings on same penguins may show if one individual has different strategies depending on the environmental conditions or food availability.

Then, why did some birds produce offshore calls during the foraging trips? The anti-predation hypothesis does not fully explain our observations of offshore calls, because predation events primarily occurred near the shore. Leopard seals (*Hydruga leptonyx*), which are one of the primary predators of gentoo penguins at our study site, mainly forage near the shore where the penguins depart for foraging trips^21, 41–43^. Moreover, we did not observe a predator or predatory situations in the videos although it could happen.

Did the offshore calls induce group association? Based on our observation that almost half of the total group associations formed within a minute after a call, the calls are likely to be related to grouping in gentoo penguins. In addition, when an individual joins a group soon after producing a call, the call can be considered as a contact call over short distances, as reported in a previous study on communication in Cape gannets^37^. We consider it unlikely that the offshore calls are sufficiently loud to act as signals over long distances. Nevertheless, it is not clear how the calls can be efficiently delivered to signal receivers under the windy environment of the open ocean. The offshore calls may not be a sole mechanism to drive group association. Although a half of group associations were observed for one minute, another half group associations did not instantly occur after a call. Also, we did not detect any behavioural changes in diving parameters (maximum dive depth, average dive depth, dive type and prey capture rate) after calling, except diving duration. These imply that unknown mechanisms may also drive group association and theses could not be exclusive with vocal behaviour.

Our data showed that group members forage at the same patch of prey (See Supplementary video S3), but we did not find clear evidence of active cooperative foraging. However, we do not exclude the possibility that sharing a prey patch at the same time could involve passive cooperative foraging^33^ and increase foraging efficiency through local enhancement^44, 45^ without intentional awareness or food calls. Information about food can be conveyed by inadvertently producing cues from feeding behaviour^44, 45^. When seabirds form larger aggregation to forage on inconspicuous prey in the open ocean, individuals may increase detectability on a food patch^46, 47^.

By deploying GPS and animal-borne cameras, Thiebault *et al*.^47^ showed that Cape gannets aggregated at food patches and that the number and frequency of foraging dives increased with the size of the aggregation. Penguins may also gain benefits from group foraging through increased foraging efficiency. Because Antarctic krill, which are the main prey of our studied gentoo penguin population (almost 99% of all prey)^34^, move in swarms, the penguins may use local enhancement to increase foraging benefits^46^. To verify the significance of local enhancement on group foraging, the effect of group size at a prey patch on group association should be determined^47^. By contrast, compared with local enhancement, collaborative foraging requires a higher level of coordination of behaviour among group members in time and place^33^. Previous research on the foraging behaviour of penguins provided evidence of coordinated behaviour, including synchronous diving, in *time* ^22, 25–27, 43^. However, because the limited angle of view of the back-borne cameras prevented us from evaluating the presence of other individuals, our results do not provide evidence that the penguins actively coordinated foraging behaviours with group members in *place*.

In summary, we have described the vocal behaviour of penguins in the open ocean, and we have discussed the possible functions of vocal communication during group behaviour. Based on our observations, we hypothesize that the group foraging behaviour of penguins may be accompanied by their vocal communication in the open ocean. Further investigations of the offshore behaviour of penguins will allow us to better understand their aquatic life. Playback experiments at foraging sites could be used to gain insight into the function of the offshore vocal signal. In addition, the placement of cameras on multiple individuals in the same group would enable a more detailed analysis of interactions among group members. Information on their interactions may enable us to better understand penguin foraging behaviour and test our hypotheses regarding the evolutionary significance of group foraging in penguins.

## Materials and Methods

### Data collection

This study was performed on a population of gentoo penguins at Narębski Point (62°14.3′S, 58°46.5′W; Antarctic Specially Protected Area No. 171) on King George Island in the South Shetland Islands during the chick-rearing period in two breeding seasons (December-January, 2014–2015 and 2015–2016). All the methods were carried out in accordance with relevant guidelines and regulations. All the experimental protocols were approved (certificate paper number: ILAD-3752 [2014.11.03] for 2014–2015 season and ILAD-3328 [2015.11.16] for 2015–2016 season) from the Korean Ministry of Foreign Affairs and Trade and according to the current laws of the Republic of Korea (‘Act on Antarctic Activities and Protection of Antarctic Environment’).

We randomly captured 26 adult gentoo penguins (19 individuals in 2014–2015, 7 individuals in 2015–2016) that had just left the nest to depart to the ocean. We deployed an animal-borne video camera (DVL400L, 143.5-mm length, 23-mm diameter, 110 g; Little Leonardo Corporation, Tokyo, Japan) on the dorsal area and two depth-acceleration recorders (ORI400-D3GT, 45-mm length, 12-mm diameter, 9 g; Little Leonardo Corporation, Tokyo, Japan) on the head and dorsum of each individual. The depth-acceleration recorders measured acceleration on a three-dimensional axis at 0.05-second intervals and a diving depth at 1-second intervals. The acceleration data were retained for a future analysis and are not reported herein. In the present study, we analysed the depth data obtained from the depth-acceleration recorders. All equipments were attached to the birds using waterproof tesa tape^48^.

The video cameras were capable of recording for 8 hours from the starting time, which was set before deployment, and the accelerometers were capable of recording diving patterns over an entire foraging trip. Microphones in the video-camera loggers recorded sound at a sampling frequency of 44.1 kHz, and the frequency response was flat within the range of 0–20,000 Hz.

Among the 26 individuals equipped with video cameras, 10 individuals exhibited offshore calls (4 individuals in 2014–2015 and 6 individuals in 2015–2016). 7 individual did not produce offshore calls (5 individuals in 2014–2015 and 2 individuals in 2015–2016). Among the 9 individuals, five did not go on a foraging trip during the video-recording time and one missed data due to unknown technical problem. Three individuals yielded very low recording quality with unknown noises were also excluded from analysis.

### Data analysis

We determined the presence of calls by watching the video files. The calls were viewed as spectrograms (256-point fast Fourier transform, overlap 50%, Hanning window) using RavenPro software (version 1.5; Cornell Laboratory of Ornithology, Ithaca, New York, USA). The time of day for each call was calculated by taking into account the starting time of each camera. The sound files were filtered with a high-pass filter above 300 Hz. Two call characteristics were measured by using RavenPro: call duration (CD) and dominant frequency (DF). CD is the length of the call and DF is the frequency with the most energy (Fig. 1).

We evaluated two types of behavioural context within three minutes before and after a calling bout occurred: group association and foraging. The three minutes of observation time was selected based on the average diving duration (41.4 ± 11.2 s) reported for the same gentoo penguin population by Lee *et al*.^40^. Group association was evaluated based on the presence/absence of other individuals in the camera frame, and the time was recorded. Foraging behaviour was investigated by analysing five variables calculated from the accelerometer and video data: dive duration, maximum dive depth, average dive depth, dive type, and prey capture rate. We compared the variables before and after 155 consecutive vocal activities between diving events. Depth data were analysed by using IGOR Pro software (version 6.3; Wavemetrics Inc., Lake Oswego, Oregon, USA). We excluded data for dives shallower than 5 m to minimize the effect of waves. Using the depth data, we calculated dive duration as the time elapsed between adjacent surface events (for details, see Lee *et al*.^40^). We also calculated the average dive depth from the maximum depth of each dive.

Dive type was determined based on the pattern of the depth profile during the bottom phase of a dive^49^. There are four types of dives: ‘V’ dives, ‘U’ dives, ‘ragged bottom’ dives, and ‘asymmetrical plateau’ dives. Previous studies have suggested that ‘V’ and ‘U’ dives are aimed at the exploration of a prey patch rather than foraging^49–51^. In this study, we classified the dives into two types: exploratory dives (‘V’ and ‘U’ dives) and foraging dives (‘ragged bottom’ and ‘asymmetrical’ dives).

Prey capture rate was determined by counting capture events on the video-recordings in slow motion during times when the penguins were hunting krill^34, 35^.

### Statistical analysis

All statistical analyses were performed using IBM SPSS for Windows (version 20.0; IBM Corp., Armonk, New York, USA). To determine how many types of calls are in the offshore call repertoire, we verified the normality of the CD and DF values of the calls by using the Kolmogorov-Smirnov test. Differences in dive duration, maximum dive depth, average dive depth, dive type and prey capture rate between before and after vocal behaviour were tested using a paired *t* test. For the paired *t* tests, we pooled dive data from calling individuals. We tested randomness of seven non-calling individuals by modelling the call frequency (number of calls per hour) per individual from other 10 calling penguins as a Poisson distribution. By rejecting the null hypothesis that non-calling individuals were at random, we included the individuals with absence of calling. To compare dive parameters (dive depth, maximum dive depth, average dive depth, dive type and prey capture rate) between calling and non-calling individuals, we used a student’s *t* test. For the student’s *t* test, we did not take account of individual difference^52, 53^.

## Electronic supplementary material

Supplementary video S1

Supplementary video S2

Supplementary video S3
